# Public Health Services in Southern Ireland

**Published:** 1923-04

**Authors:** 


### PUBLIC HEALTH SERVICES IN SOUTHERN IRELAND.

"THAT this meeting calls upon the Government to appoint a Ministry of Health and Public Welfare, so that the very urgent needs of the community in regard to health services may be considered and dealt with without delay," was the resolution passed with acclamation at the recent Public Health Conference in the Mansion House, Dublin. The meeting was held undes the auspices of the Irish Nurses' & Midwives' Union, 29 South Anne Street, Dublin, and the chair was taken by Miss Louie Bennett, President, who said that the Union had always felt the necessity for a constructive as well as a defensive policy. They had gained through their members a knowledge of the lack of nursing facilities in the country districts in Ireland, and had, therefore, formulated a scheme for the provision of such nurses, which had been forwarded to the Government. Since a Ministry of Health had not yet been formed, it was important that public opinion should be roused on this important subject.

### The Necessity for a Ministry of Health.

Mr. R. J. P. Mortished then outlined the proposed scheme for the provision of nurses for country districts, to be carried out under the proposed Ministry of Health. Some kind of national nursing scheme was necessary. Medical inspection of school children was provided for in an Act which was on the Statute book, but had never been enforced in Ireland. He emphasised the necessity for a Ministry of Health. Dr. T. R. Hennessy, of the Irish Medical Committee, said that the various Public Health Acts were practically inoperative in Ireland, and that the position of health services is little better in that country to-day than it was a hundred years ago. The importance of efficient Public Health services was regarded with indifference by the people of the country. Dr. Robert Rowlette, a member of the Irish Public Health Council, stated that in the whole of the twenty-six counties of the Free State there was only one whole-time Medical Officer of Health, with one other for Northern Ireland.

### Need of Child Welfare Work.

Miss Smithson said that a Cottage Hospital, or even a house where two or three nurses could live together, could be made a centre of light and learning in health matters to the surrounding country. She also thought a Child Welfare Scheme should be started by every municipality.

